# Comparing five‐factor personality traits and psychological distress between dysphonic patients and individuals with vocally healthy voices

**DOI:** 10.1002/brb3.3641

**Published:** 2024-08-05

**Authors:** Mahshid Aghajanzadeh, Payman Dabirmoghaddam, Mehdi Soleimani, Saeed Saeedi

**Affiliations:** ^1^ Department of Speech Therapy, School of Rehabilitation Tehran University of Medical Sciences Tehran Iran; ^2^ Otorhinolaryngology Research Center Tehran University of Medical Sciences Tehran Iran; ^3^ Department of Psychiatry, School of Medicine Tehran University of Medical Sciences Tehran Iran

**Keywords:** dysphonia, five‐factor personality traits, psychological distress, voice

## Abstract

**Introduction:**

It was reported that voice can carry information about personality and psychological distress. In the current study, the relationship between five‐factor personality traits and psychological distress with voice was enlightened from diverse aspects.

**Methods:**

A total of 119 participants (55 with and 64 without dysphonia) sustained vowels /a/ and /i/, read six standard sentences, and answered a question. Three raters auditory–perceptually evaluated the vocal samples using the Persian version of CAPE‐V. The participants were distributed into four groups (vocally healthy, mild, moderate, and severe dysphonia). They completed two questionnaires: NEO Five‐Factor Inventory (NEO‐FFI) and Depression, Anxiety, and Stress Scale‐21.

**Results:**

Results showed that the conscientiousness (*U* = 1146.500, *z* = −3.27, *p* = .001) in the dysphonia group was significantly less than the vocally healthy group. Depression (*U* = 1381.000, *z* = −2.03, *p* = .042) and anxiety (*U* = 1181.000, *z* = −3.10, *p* = .002) in the dysphonia group were significantly higher than in the vocally healthy group. In comparing different abnormal overall voice qualities, the mild dysphonia group revealed significantly lower conscientiousness (*p* = .001) and significantly higher anxiety (*p* = .002) relative to the vocally healthy group.

**Conclusions:**

Findings indicated that the conscientiousness trait could play an influential role in persons with dysphonia and its psychological status. The voice care team should consider conscientiousness and psychological distress during the assessment and treatment of dysphonic patients.

## Graphical abstract

There appears to be a potential relationship between the conscientiousness trait of personality, depression, and anxiety levels with the voice signals sourced from the larynx. People with voice disorders, known as dysphonia, exhibited lower levels of conscientiousness, elevated levels of depression, and increased levels of anxiety in contrast to normal controls.

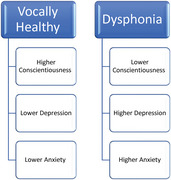

## 1 INTRODUCTION

Personality is defined as one's patterns of behaviors, feelings, and thoughts over the course of time and across different circumstances (Twenge & Campbell, 2016). Personality can be conceptualized as a structure with higher order and lower order traits. Although higher order dimensions present a holistic image of personality, more details are obtainable with the lower order traits (J. M. Lee et al., 2021). It has been postulated that personality traits may influence physical and mental health status and also impose some impairments on physical functioning after developing illnesses (Goodwin & Friedman, 2006).

A personality model, the five‐factor model, has been suggested, which is composed of five higher order traits: extraversion, neuroticism, agreeableness, openness, and conscientiousness (Costa & McCrae, 2008). Extraversion assesses the demonstration of feelings, intimacy, accompanying others, controlling or dictating others' behavior (social dominance), daily activities, excitement, and happiness (Levallius et al., 2015). Neuroticism indicates anxiety, anger, depression, self‐consciousness (susceptibility to difficult social circumstances, e.g., teasing), impulsiveness (abiding disappointment and controlling needs), and handling stress (Levallius et al., 2015). People with high levels of neuroticism react inappropriately to environmental stress and have tendency to interpret normal situations as intimidating (Widiger & Oltmanns, 2017). Agreeableness evaluates the degree of trust in people, honesty, altruism, abstaining showing anger, humbleness, and sympathy (Levallius et al., 2015). Individuals with high agreeableness are more collaborative, trustful, and compassionate (Twenge & Campbell, 2016). Openness reflects imagination, enjoyment of beauty, accepting the experienced feelings, creativity, flexibility to new opinions, and considering the possible change in previous standards (Chamorro‐Premuzic & Furnham, 2005; J. M. Lee et al., 2021; Levallius et al., 2015). Conscientiousness measures beliefs about dealing with difficult situations, discipline, and tidiness; relying on morality, reaching targets, and doing a series of actions regardless of tedium; and thinking ahead of taking actions (Levallius et al., 2015). Conscientiousness predicts one's successful function in educational, occupational, financial, managing, and marital aspects of life (Dudley et al., 2006; Judge et al., 2002; Moffitt et al., 2011; Noftle & Robins, 2007; Roberts & Bogg, 2004).

It was found that people with certain personality traits are at greater risk of developing chronic diseases such as chronic pulmonary disease, cerebrovascular disease, diabetes, and so on (Sutin et al., 2013). Among the five‐factor personality traits, conscientiousness and neuroticism have been the key influencers of health. It was discovered that individuals who reported having physical illnesses exhibited fewer restrictions in physical exertion if they possessed high levels of conscientiousness, as opposed to those individuals with low levels of conscientiousness (Goodwin & Friedman, 2006). In another study, conscientiousness and neuroticism were significant predictors of patient mortality, with higher neuroticism scores associated with a 37.5% higher mortality rate and lower conscientiousness scores linked to a 36.4% increased mortality rate (Christensen et al., 2002).

Dysphonia or voice disorder is defined as any alteration in the quality, pitch, or loudness of someone's voice compared to his/her peers with alike age, sex, region, and culture (Stemple et al., 2020). It is estimated that around 3%–10% of the general population suffer from dysphonia (Rubin et al., 2003). According to a new survey, around 20% of Americans have experienced dysphonia at some stage in their lives. Additionally, 12.6% of respondents indicated that they are currently dealing with dysphonia, while 9.5% reported experiencing recurring dysphonia (Huston et al., 2024). In Sweden, it was found that 16.9% of the population experienced voice problems. Females were noted to have a higher prevalence of voice issues compared to males, and individuals over the age of 65 were significantly more likely to experience voice problems (Lyberg‐Åhlander et al., 2019).

The larynx as a musculo–cartilaginous structure in the body has a noticeable role in controlling emotional expression. The larynx is responsible for expressing various emotions such as fear, anger, sadness, and joy through the voice. By the way, the voice is a uniquely human characteristic showing interpersonal differences between people; it reflects some information about personality (Stemple et al., 2020). Goodwin and Friedman (2006) found that people with higher scores in neuroticism and lower scores in conscientiousness had an increased rate of mental and physical disorders (Goodwin & Friedman, 2006). Roy et al. found that neuroticism and extraversion traits in patients with vocal nodules and functional dysphonia were different compared with vocally healthy, spasmodic dysphonia, and unilateral vocal fold paralysis groups. Patients diagnosed with vocal nodules exhibited extroverted traits, whereas those with functional dysphonia displayed introverted characteristics, along with elevated levels of neuroticism. Therefore, the trait theory of voice disorders was proposed, which is indicative of the effects of personality traits on dysphonia development (Roy et al., 2000a, 2000b). The researchers concluded that personality examination has a leading role in the prevention and treatment of dysphonia (Roy et al., 2000b; Roy & Bless, 2000). A systematic review has indicated that combining cognitive behavioral therapy (CBT), which is an organized approach for addressing the issues faced by clients in order to alleviate symptoms associated with a range of mental health disorders, with a primary focus on depression and anxiety with traditional voice therapy for functional voice disorders can lead to various advantages such as enhanced voice quality, improved psychosocial well‐being, and reduced risk of relapse. It is viable to provide training in CBT‐enhanced voice therapy to speech–language pathologists (SLPs) (Gray et al., 2021).

Various personality traits and psychological distress, such as agreeableness, conscientiousness, depression, and anxiety, can influence the acoustic measurements of voice signals (Saeedi et al., 2023). Interestingly, the effects of personality on voice pathology are not limited to just dysphonic patients. The results of an investigation highlighted the role of SLP's personality and cognitive emotion regulation on the accuracy of their auditory–perceptual analysis for dysphonic patients. When certain maladaptive elements of personality and cognitive emotion regulation were on the rise, while adaptive elements were declining, the perceptions of grade and strain of the voice samples perceived by SLPs became increasingly unfavorable. These findings implied the potential presence of additional confounding variables beyond the main factors, such as the SLPs' training and experience in perceptual voice assessment (Alemdağ et al., 2023).

In addition to certain personality traits, the presence of psychopathologies (psychological distress), such as depression, anxiety, and so on, was reported in voice dysphonic patients (Mirza et al., 2003). The interesting point is that the dysphonia type (organic, neurogenic, and functional) was known effective in the prevalence rate of psychopathologies; the patients with vocal fold paralysis showed more psychopathologies in comparison with patients with functional dysphonia and spasmodic dysphonia (Mirza et al., 2003). According to research, dysphonic patients with benign lesions who had lower level of anxiety were successfully treated; on the other hand, more anxious patients were resistant to treatment (Ng et al., 2013). Likewise, in another study, the researchers concluded that perceived stress in addition to hyperactivity and impulsiveness, which are related to neuroticism, are key factors of vocal fold nodules development (Abeida et al., 2013). In a study, it was observed that dysphonia led to psychological distress, often exacerbating the perception of the severity of vocal symptoms by patients as a result. Most patients had a positive outlook toward speech therapy, but a few struggled to make consistent progress. The perceived sense of control, in terms of managing the dysphonia or their emotional responses to it, appeared to influence whether their reactions were beneficial or detrimental (Misono et al., 2019).

In this paper, the differences between personality traits and psychological distress and dysphonia were compared in the Persian population using two well‐validated questionnaires. Also, the effects of dysphonia severity and dysphonia types (organic, neurogenic, and functional) on these differences were examined.

## 2 MATERIALS AND METHODS

### 2.1 Participants

The data used in this study were obtained from the specific dataset mentioned in the previous publication, where a comprehensive analysis was conducted to explore the relationship between acoustic measurements and personality and psychological distress (Saeedi et al., 2023). The power analysis formula was used to determine the necessary sample size (Chow et al., 2017). The formula took into account parameters such as the standard deviation of the dysphonia group (7.4), the standard deviation of the control group (4.1), *z*_∝⁄2_ (2.58) at a 99% confidence level (*α* = .01), *z_β_* (0.85) at an 80% test power, and a clinically significant mean difference (*δ*) of 13 (with a 50% error probability). These means and standard deviations were based on a previous study (Willinger et al., 2005). It was established that a sample size of 64 would be necessary for each group, and the study recruited a total of 128 adults for participation. Within the dysphonia group, 54.5% of individuals were male and 45.5% were female. Conversely, the vocally healthy group consisted predominantly of females, making up 68.8% of the participants. The descriptive statistics of demographics (gender and age) and clinical characteristics (abnormal overall voice quality and dysphonia type) are reported in Table 1.

**TABLE 1:** Participant demographics and clinical characteristics (*n* = 119).

Characteristics data			
**Gender**			
Male		50 (42%)	
Female		69 (58%)	
**Age (years)**			
Average (SD)		39.73 (11.82)	
Median		38	
Range		18–65	
**Abnormal overall voice quality**			
Vocally healthy		64 (53.80%)	
Dysphonia	Mild	55 (46.20%)	24 (20.20%)
	Moderate		23 (19.30%)
	Severe		8 (6.70%)
**Dysphonia type**			
Organic		33 (60.00%)	
Neurogenic		10 (18.20%)	
Functional		12 (21.80%)	

Abbreviation: SD, standard deviation.

The research was conducted at the head and neck clinic of Amir Alam Hospital, Tehran, Iran, in the period from July 2021 to October 2021. The authors assert that all procedures contributing to this work comply with the ethical standards of the relevant national and institutional committees on human experimentation and with the Helsinki Declaration, as the latest revised in 2008 (Puri et al., 2009). All the procedures were approved by Research Ethics Committees of School of Nursing and Midwifery and Rehabilitation‐Tehran University of Medical Sciences (approval ID: IR.TUMS.FNM.REC.1399.162). Written informed consent was received from all the participants.

### 2.2 Inclusion criteria

All the participants had to have these criteria to qualify for participation in the study: (a) age range of 18–65 years, (b) no hearing impairment, (c) no cognitive disorder, (d) no history of voice therapy, and (e) not taking any psychotropic medications (e.g., antidepressants). The dysphonic patients had to have abnormal overall voice quality in the auditory–perceptual assessment. The voice of the control group, including the volunteer patients’ family members and the staff at the hospital had to be auditory–perceptually confirmed as vocally healthy. This group had to have no self‐reported voice complaint, no neurologic disorder, no communication disorder, no history of head and neck surgery, and no upper respiratory tract infection (in the last 3 weeks before the sampling).

### 2.3 Medical case history and voice self‐assessment

To verify the inclusion criteria and collect essential data, all participants were provided with a researcher‐developed questionnaire in printed form. This questionnaire consisted of sections covering demographic details, medical background, and history of voice issues. As self‐evaluations are now widely used in voice clinics and research as they offer important information from the patient's perspective about their condition (Branski et al., 2010), within the last section dedicated to voice self‐assessment, participants were required to rate their overall vocal hoarseness on a scale from 0 (normal voice) to 5 (severe hoarseness). There was no specified time limit for filling out the questionnaire (Stemple et al., 2020). If necessary, an SLP would provide clarification on any ambiguous items orally.

### 2.4 Auditory–perceptual evaluation

The Persian validated version of the Consensus Auditory‐Perceptual Evaluation (ATSHA) was given to each participant (Khoramshahi et al., 2018). Sitting on a comfortable chair, they were instructed to sustain vowels /a/ and /i/ for about 3−5 s at their comfortable pitch and loudness, read six sentences, and respond to a question (“Tell me about your voice problems”). The voice recordings were obtained by employing a Zoom H5 Handy Recorder (Zoom Corporation) positioned at a fixed distance of 10 cm and an angle of 45° from the participant's mouth. This setup was established on a pedestal within a soundproof booth, ensuring that the ambient noise level remained below 38 decibels (Patel et al., 2018). The recorder's sampling rate and quantization were set at 44,100 Hz and 16 bit, respectively. After the voice samples were recorded, two qualified SLPs, with over 5 years of clinical experience in voice therapy, separately listened to the voice samples wearing headphones AKG K612 PRO (AKG Acoustics). Notably, they were blinded to the demographic characteristics and the outcomes of other conducted assessments, such as videolaryngostroboscopy imaging. Overall severity (OS) rating of the ATSHA was utilized (Khoramshahi et al., 2018). The ranges of OS < 10, 10 ≤ OS < 40, 40 ≤ OS < 70, and OS ≥ 70 were regarded to distinguish participants in vocally healthy, mild, moderate, and severe dysphonia groups, respectively (Mizuta et al., 2020). Two judges’ scores were averaged. Supposing the disagreement of the first two judges in the group determination of a specific voice sample, the third SLP who also had over 5 years of clinical experience in voice therapy was called to evaluate, and then the mean of three scores was selected as the final decision.

### 2.5 Videolaryngostroboscopy

Videolaryngostroboscopy imaging has a prominent function in the assessment and treatment of dysphonia (Mehta & Hillman, 2012). EndoSTROBE system (KARL STORZ) with a 70° rigid endoscope (KARL STORZ‐ENDOSKOPE pulsar stroboscopy system 20140020) was used in this study. The dysphonic patients with variant degrees of severity (mild, moderate, and severe) were invited to sit on a chair with their backs in the videolaryngostroboscopy section of the hospital. They were asked to lean forward while opening their mouth and sustain the vowel /i/ with both normal and high pitch.

### 2.6 Medical diagnosis of dysphonic patients

Upon analyzing the outcomes from different sections of assessment such as medical case history, voice self‐assessment, auditory‐perceptual voice assessment, and videolaryngostroboscopy, the patient's specific pathology was diagnosed and subsequently categorized into one of the etiologic groups: organic, neurogenic, and functional by an otorhinolaryngologist and an SLP, each having 5 years of experience in voice pathology. Organic voice disorders are associated with structural abnormalities in the vocal tract, such as the lungs, respiratory muscles, larynx, pharynx, and oral cavity, or with diseases affecting specific structures within the vocal tract. On the other hand, neurogenic voice disorders are linked to neurological conditions that result in impaired vocal fold closure due to paralysis, weakness, or neurological diseases. In contrast, functional voice disorders do not have an organic or neurological basis. Despite the physical capability of the mechanisms involved in respiration, phonation, and resonance to support normal voice production, there is a lack of proper functional balance (Boone et al., 2020).

### 2.7 Questionnaires

NEO–Five‐Factor Inventory (NEO–FFI) is a shorter version of the NEO Personality Inventory‐Revised questionnaire, which measures the five principal personality traits (neuroticism, extraversion, openness to experience, agreeableness, and conscientiousness). Sixty items of NEO–FFI are rated on a Likert‐based scale ranging from 1 (“strongly disagree”) to 5 (“strongly agree”) (Costa & McCrae, 2008). The internal consistency of Persian‐validated version of NEO–FFI was in the range of 0.54 (openness) to 0.79 (neuroticism) (Soleimani et al., 2022). Depression, Anxiety and Stress Scale‐21 (DASS‐21) is the compact version of DASS, which measures depression, anxiety, and stress among adults (Bibi et al., 2020). It is noteworthy that depression, anxiety, and stress are considered as an indicator of psychological distress (Viertiö et al., 2021). Twenty‐one items of DASS‐21 are rated on a Likert‐based scale from 0 (“did not apply to me at all‐never”) to 3 (“applied to me very much, or most of the time‐almost always”) (Bibi et al., 2020). The internal consistency of the Persian‐validated version of DASS‐21 for each subscale was 0.77 (depression), 0.79 (anxiety), and 0.78 (stress) (Soleimani et al., 2015). Persian versions of NEO‐FFI and DASS‐21 were given to participants to fill in. No time constraints were considered for participants to fill in these questionnaires. To minimize the bias, all NEO‐FFI and DASS‐21 questionnaires were reviewed on account of suspicious responding patterns (e.g., selecting only one answer for all the questions), which showed that participants have answered the questions with disinterest or not careful consideration. Suspicious response patterns were identified in nine questionnaires of the dysphonic group; these participants were excluded from the study.

### 2.8 Statistical analysis

Statistical analysis was run via the software SPSS version 25 (SPSS Corp). An assessment of inter‐rater reliability of auditory–perceptual assessment between judges was conducted by employing the kappa statistic to ascertain the level of agreement among two raters. The numerical intervals 0–0.20, 0.21–0.40, 0.41–0.60, 0.61–0.80, and 0.81–1.00 were defined as slight, fair, moderate, substantial, and almost perfect agreement between judges, respectively (Nichols et al., 2010). The Kolmogorov–Smirnov test was performed to determine the normality distribution of personality traits and psychological distress. On the condition of normal distribution, the independent sample *t* test was used to compare the personality traits and psychological distress means between vocally healthy and dysphonia groups. In the case of non‐normal distribution, the Mann–Whitney *U* test was applied. On the assumption of the normal distributions, the one‐way factorial analysis of variance test followed by the Tukey's honestly significant difference post hoc test was used to compare the means of personality traits and psychological distress between different levels of abnormal overall voice quality groups and between different types of dysphonia groups. In the case of a non‐normal distribution, the nonparametric Kruskal–Wallis *H* test followed by Dunn's pairwise post hoc test was employed. The significance level for all the other statistical tests was considered at 0.05. To remove the influence of sample size on the outcomes, the effect size was also calculated (E. Lee et al., 2014; McCough & Faraone, 2009). Hedges’ *g*, correlation coefficient, partial eta‐squared, and epsilon‐squared were used to calculate the effect size for independent sample *t*, Mann–Whitney *U*, analysis of variance, and Kruskal–Wallis *H* tests, respectively (Table 2) (Tomczak & Tomczak, 2014). In order to explore the relationship between personality traits and psychological distress with each other, and their relationship with age, the Pearson correlation coefficient and Spearman's rank correlation coefficient were employed. To assess the strength of the correlation coefficient effect size, recommended thresholds for the social sciences were utilized: 0.10−0.29 indicating a small effect, 0.30−0.49 representing a medium effect, and 0.50−1.00 signifying a large effect (Rodijk et al., 2022).

**TABLE 2:** The interpretation of effect size strength.

Effect size index	Effect size strength		
	Small	Moderate	Large
Hedges’ *g*	0.2	0.5	0.8
Correlation coefficient	0.1	0.3	0.5
Partial eta‐squared	0.01	0.06	0.14
Epsilon‐squared	0.01	0.04	0.16

## 3 RESULTS

The inter‐rater reliability among the two raters in auditory–perceptual assessment using OS of ATSHA yielded a kappa value of 0.72 (*p* = .000), with a 95% confidence interval ranging from 0.62 to 0.82.

The results showed that mean scores of conscientiousness (*U* = 1146.500, *z* = −3.27, *p* = .001), depression (*U* = 1381.000, *z* = −2.03, *p* = .042), and anxiety (*U* = 1181.000, *z* = −3.10, *p* = .002) were significantly different between the vocally healthy and dysphonia groups. In comparison to the vocally healthy group, the dysphonia group had lower mean scores of conscientiousness and higher mean scores of depression and anxiety (Table 3).

**TABLE 3:** Comparison of personality traits and psychological distress between the vocally healthy and dysphonia groups (*n* = 119).

Variable	Vocally healthy	Dysphonia	Test statistic	*p* value	Effect size (*r*)
	Mean (SD)	Mean (SD)			
Extraversion	31.76 (5.16)	30.41 (6.75)	1562.000^a^	.290	0.096^b^
Neuroticism	18.92 (4.15)	20.78 (6.41)	1.843^c^	.069	0.350^d^
Agreeableness	31.57 (4.48)	30.50 (4.78)	1.256^c^	.212	0.231^d^
Openness	26.68 (5.52)	25.10 (4.00)	1424.000^a^	.073	0.164^b^
Conscientiousness	39.60 (5.10)	36.03 (5.88)	1146.500^a^	.001^*^	**0.300** ^b^
Depression	3.04 (3.05)	4.27 (3.75)	1381.000^a^	.042^*^	0.186^b^
Anxiety	3.10 (2.72)	4.85 (3.42)	1181.000^a^	.002^*^	0.284^b^
Stress	5.82 (3.16)	6.78 (4.25)	1591.000^a^	.365	0.082^b^

Abbreviation: SD, standard deviation.

^a^ Mann–Whitney *U* value.

^b^ Correlation coefficient

^c^ *t* value.

^d^ Hedges’ *g*.

^*^Statistical significance at *p* < .05.

The dysphonia group of females showed significantly lower conscientiousness (*t* (67) = −2.70, *p* = .009) and higher anxiety (*U* = 327.500, *z* = −2.79, *p* = .005) compared with the vocally healthy (Table 4).

**TABLE 4:** Comparison of personality traits and psychological features between normal voice and dysphonia groups by gender (*n* = 119).

Variable		Normal voice	Dysphonia	Test statistic	*p* value	95% Confidence interval of the difference	
		Mean (SD)	Mean (SD)				
						Lower	Upper
Extraversion	Male	32.35 (5.60)	31.36 (6.16)	0.573^a^	.569	−2.24	3.91
	Female	31.50 (4.99)	29.28 (7.36)	1.48^a^	.141	−0.34	6.11
Neuroticism	Male	19.20 (5.00)	20.03 (5.49)	0.544^a^	.589	−2.46	4.43
	Female	18.79 (3.76)	21.68 (7.38)	1.82^a^	.078	−0.75	−5.19
Agreeableness	Male	30.40 (4.50)	30.66 (3.00)	0.252^a^	.802	−3.29	2.69
	Female	32.11 (4.43)	30.32 (6.36)	1.37^a^	.174	−0.71	4.12
Openness	Male	24.80 (5.67)	24.50 (4.05)	0.204^a^	.839	−1.86	2.39
	Female	27.54 (5.30)	25.84 (3.89)	1.40^a^	.165	−0.81	4.39
Conscientiousness	Male	38.25 (5.07)	35.50 (6.17)	1.65^a^	.105	−6.09	0.59
	Female	40.22 (5.04)	36.68 (5.56)	−2.70^a^	.009*	−6.16	−0.92
Depression	Male	3.40 (2.81)	4.26 (2.75)	244.50^b^	.268	–	–
	Female	2.88 (3.17)	4.28 (4.74)	454.00^b^	.225	–	–
Anxiety	Male	3.20 (2.89)	4.23 (2.58)	222.00^b^	.120	–	–
	Female	3.06 (2.67)	5.60 (4.15)	327.00^b^	.005*	–	–
Stress	Male	5.60 (3.18)	5.70 (3.18)	0.109^a^	.914	−1.74	1.94
	Female	5.93 (3.17)	8.08 (5.01)	1.93^a^	.061	−0.10	4.40

Abbreviation: SD, standard deviation.

^a^ *t* value.

^b^ Mann–Whitney *U* value.

*Statistical significance at *p* < .05

Based on the results of the correlation analysis, it was found that there was no statistically significant relationship between any of the personality traits and psychological distress with age within the entire sample (Table 5).

**TABLE 5:** Correlation between personality traits and psychological distress with age (*n* = 119).

		Extraversion	Neuroticism	Agreeableness	Openness	Conscientiousness	Depression	Anxiety	Stress
**Age**	***r***	0.077^a^	0.003^b^	0.098^a^	−0.134^b^	0.022^a^	0.043^a^	0.030^a^	0.151−^a^
	***p* value**	.406	.974	.290	.146	.810	.640	.845	.101

^a^ Pearson correlation coefficient.

^b^ Spearman's rank correlation coefficient.

*Statistical significance at *p *< .05.

The results of Kruskal–Wallis *H* showed that the difference between conscientiousness (*H *(3) = 14.97, *p* = .002) and anxiety (*H *(3) = 11.02, *p* = .012) was statistically significant between at least two groups of abnormal overall voice quality (Table 6). The following Dunn's pairwise post hoc test for conscientiousness (*p* = .001) and anxiety (*p* = .002) showed that the median difference was statistically significant between the vocally healthy and mild dysphonia groups. The vocally healthy group exhibited elevated levels of conscientiousness and reduced levels of anxiety in contrast to the group with mild dysphonia.

**TABLE 6:** Comparison of personality traits and psychological distress between different levels of abnormal overall voice quality (*n* = 119).

Variable	Vocally healthy	Mild dysphonia	Moderate dysphonia	Severe dysphonia	Test statistic	*p* value	Effect size (*r*)
Extraversion	31.76 (5.16)	28.62 (7.16)	31.43 (6.34)	32.87 (5.93)	2.292^a^	.514	0.019^b^
Neuroticism	18.92 (4.15)	22.25 (6.48)	20.08 (5.72)	18.37 (7.81)	2.533^c^	.060	**0.062** ^d^
Agreeableness	31.57 (4.48)	29.75 (5.12)	31.21 (4.22)	30.75 (5.54)	3.503^a^	.320	0.029^b^
Openness	26.68 (5.52)	25.16 (4.62)	25.08 (3.18)	25.00 (4.62)	3.424^a^	.331	0.028^b^
Conscientiousness	39.60 (5.10)	33.87 (6.69)	37.43 (4.30)	38.50 (5.68)	14.971^a^	.002*	**0.126** ^b^
Depression	3.04 (3.05)	5.25 (4.51)	3.78 (3.14)	2.75 (1.98)	6.164^a^	.104	**0.052** ^b^
Anxiety	3.10 (2.72)	5.66 (3.88)	4.30 (3.09)	4.00 (2.56)	11.028^a^	.012*	**0.093** ^b^
Stress	5.82 (3.16)	8.00 (5.06)	5.91 (3.41)	5.62 (3.06)	3.296^a^	.348	0.027^b^

^a^ Kruskal–Wallis *H* value.

^b^ Epsilon‐squared.

^c^ *F* value (analysis of variance one‐way).

^d^ Partial eta‐squared.

*Statistical significance at *p* < .05.

The results of Kruskal–Wallis *H* showed that the difference between conscientiousness (*H *(3) = 12.64, *p* = .005) and anxiety (*H* (3) = 13.18, *p* = .004) was statistically significant between at least two groups of dysphonia types (Table 7). The following Dunn's pairwise post hoc test showed that the median of conscientiousness was statistically significant between the vocally healthy and organic functional dysphonia groups (*p* = .004) and between vocally healthy and functional dysphonia groups (*p* = .007). The vocally healthy group demonstrated elevated levels of conscientiousness in comparison to the groups experiencing organic and functional dysphonia. The following Dunn's pairwise post hoc test also showed that the median of anxiety was statistically significant between the vocally healthy and functional dysphonia groups (*p* = .365). The levels of anxiety were found to be higher in both the functional dysphonia group when compared to the vocally healthy group.

**TABLE 7:** Comparison of personality traits and psychological distress between different dysphonia types (*n* = 119).

Variable	Vocally healthy	Organic dysphonia	Neurogenic dysphonia	Functional dysphonia	Test statistic	*p* value	Effect size (*r*)
Extraversion	31.76 (5.16)	30.33 (7.11)	30.80 (6.23)	30.33 (6.66)	1.731^a^	.630	0.014^b^
Neuroticism	18.92 (4.15)	20.69 (5.54)	19.30 (6.34)	22.25 (6.34)	1.767^c^	.157	0.044^d^
Agreeableness	31.57 (4.48)	30.18 (4.27)	31.50 (6.70)	30.58 (4.62)	0.726^c^	.539	0.019^d^
Openness	26.68 (5.52)	25.42 (3.92)	24.60 (3.83)	24.66 (4.57)	3.467^a^	.325	0.029^b^
Conscientiousness	39.60 (5.10)	35.57 (6.32)	38.50 (4.35)	35.25 (5.57)	12.641^a^	.005*	**0.107** ^b^
Depression	3.04 (3.05)	4.57 (4.01)	3.40 (3.33)	4.16 (3.48)	4.842^a^	.184	**0.041** ^b^
Anxiety	3.10 (2.72)	4.27 (3.40)	5.50 (3.59)	5.91 (3.26)	13.181^a^	.004*	**0.111** ^b^
Stress	5.82 (3.16)	6.51 (4.37)	7.20 (4.15)	7.16 (4.28)	1.779^a^	.620	0.015^b^

^a^ Kruskal–Wallis *H* value.

^b^ Epsilon‐squared.

^c^ *F* value (analysis of variance one‐way).

^d^ Partial eta‐squared.

*Statistical significance at *p* < .05.

It is possible that the group comparisons (vocally healthy, mild, moderate, severe and vocally healthy, organic, neurogenic, functional dysphonia) are insufficiently powered given the smaller sample sizes of these groups.

The results of Spearman's rank correlation coefficient showed that there was a small inverse correlation between agreeableness with anxiety (*r* (117)* = *−0.239, *p* = .009) and stress (*r* (117) *= *−0.243, *p* = .008), and between conscientiousness with anxiety (*r* (117) *= *−0.289, *p* = .001) and stress (*r* (117)* = *−0.225, *p* = .014). There is a medium inverse correlation between extraversion with anxiety (*r* (117) *= *−0.316, *p* = .000) and stress (*r* (117)* = *−0.461, *p* = .000), between agreeableness with depression (*r* (117)* = *−0.368, *p* = .000), and between conscientiousness with depression (*r* (117)* = *−0.397, *p* = .000). Finally, there is a large inverse correlation between extraversion with depression (*r* (117)* = *−0.519, *p* = .000), and a large positive correlation between neuroticism with depression (*r* (117)* = *0.522, *p* = .000), anxiety (*r* (117)* = *0.553, *p* = .000), and stress (*r* (117)* = *0.606, *p* = .000) (Table 8).

**TABLE 8:** Correlation between personality traits with psychological distress (*n* = 119).

		Depression	Anxiety	Stress
Extraversion	*r*	−0.519^a^	−0.316^a^	−0.461^a^
	*p* value	.000	.000	.000
Neuroticism	*r*	0.522^a^	0.553^a^	0.606^a^
	*p* value	.000	.000	.000
Agreeableness	*r*	−0.368^a^	−0.239^a^	−0.243^a^
	*p* value	.000	.009	.008
Openness	*r*	−0.134^a^	0.051^a^	0.123^a^
	*p* value	.145	.584	.184
Conscientiousness	*r*	−0.397^a^	−0.289^a^	−0.225^a^
	*p* value	.000	.001	.014

^a^ Spearman's rank correlation coefficient.

*Statistical significance at *p *< .05.

## 4 DISCUSSION

The role of personality and psychological distress as one of the etiologic factors of dysphonia or as secondary symptoms of dysphonia has been disputed for a long time (Roy et al., 2000b). This study sought to answer whether certain personality traits and psychological distress differ significantly between the Persian‐speaking participants with/without dysphonia.

Our current finding supports the view that neuroticism as a basic personality trait does not differ between the two groups. This result is congruent with the past studies (Kasefy et al., 2020; Toles et al., 2021). There was not a significant difference in another lower order trait of neuroticism, stress, between the normal voice and dysphonia groups.

Extraversion scores were not significantly different in dysphonia and the vocally healthy groups. This result is in line with the past study (Kasefy et al., 2020; J. M. Lee et al., 2021; Toles et al., 2021). As was shown previously, extraverts speak more than usual with/without increased intensity, so they are more at risk of phonotrauma (Toles et al., 2021). The openness mean difference between the vocally healthy and dysphonia groups was not significantly different. This finding agrees with the previous research (Kasefy et al., 2020; Lee et al., 2021). Agreeableness is able to hypothetically impact the dysphonic patient's relationship with the voice therapist and his/her adherence to the therapeutic process (Toles et al., 2021). There was no significant difference in agreeableness scores between the vocally healthy and dysphonia groups. This result is congruent with the previous research study (Kasefy et al., 2020; Lee et al., 2021).

People with higher conscientiousness levels are more purposeful, systematic, dutiful, and hardworking. These people are expected to be superior in self‐control, self‐regulation, and have a greater tendency to engage in health‐promoting behaviors like exercising, avoiding smoking, and substance use (Goodwin & Friedman, 2006). Self‐control is linked well with avoidance of unacceptable behaviors, such as drug abuse, and is a key element of optimal health (Moffitt et al., 2011). As a result, people with higher conscientiousness levels cope better with the challenges and problems of their lives and therefore experience less anxiety and depression (Hoyle, 2010). As people with lower conscientiousness levels have a hedonistic tendency and impulsivity, they might engage in leisure activities accompanied by abusive vocal behaviors, such as shouting or loud talking that may cause phonotrauma and consequently dysphonia (Cervone & Pervin, 2019). In this study, the dysphonia group had significantly lower scores of conscientiousness than the vocally healthy group. This result is consistent with the past study done by on children (J. M. Lee et al., 2021). This result was in contradiction with the previous research. This conflict might be due to the smaller sample size of the mentioned study relative to ours (Kasefy et al., 2020).

The impact of gender on personality traits and psychological distress between vocally healthy and dysphonia groups was observed primarily in conscientiousness and anxiety levels. Notably, female participants in the dysphonia group in the study exhibited significantly lower conscientiousness and higher anxiety compared to their counterparts in the vocally healthy group. This outcome aligns well with the previous research that has highlighted the female gender as a potential risk factor for experiencing depression and anxiety within both populations (Dietrich et al., 2008). This suggests that females may be more susceptible to psychological challenges than their male counterparts. Interestingly, aside from psychological distress, there is a gap in the existing literature regarding the direct influence of gender on the personality traits of individuals in the vocally healthy and dysphonia groups. Therefore, it is recommended that future studies explore the role of gender in greater depth to gain a better understanding of its implications.

It was found that there is no significant relationship between age and personality traits and psychological distress in the entire sample, aligning with previous research that also found no link between age and psychological distress (Zhang et al., 2020). With limited evidence on the potential impact of age on personality traits and psychological distress in the dysphonic population, it is advisable for additional research to be conducted.

When examining various personality traits and psychological distress variables in relation to abnormal overall voice quality, it was found that only conscientiousness and anxiety showed significant differences. The group of vocally healthy individuals displayed higher levels of conscientiousness and lower levels of anxiety compared to the group experiencing mild dysphonia. However, no significant differences were observed between the mild, moderate, and severe levels of abnormal overall voice quality, which implies that all the dysphonic patients have a similar profile in terms of personality traits and psychological distress. This result might be due to the intricate links between abnormal overall voice quality with duration of dysphonia and reduced level of voice‐related quality of life (Cohen, 2010). As the dysphonia persists for a longer time, individuals with dysphonia believe that their symptoms will worsen, resulting in them becoming less responsive to treatment (Buck, 2007). Also, perhaps this finding could be linked to the adaptation phenomenon; over time, dysphonia may become habitual for the individual manifesting less psychological distress. To the best of the authors' knowledge, no research has yet investigated the relationship between abnormal overall voice quality with personality traits and psychological distress. Thus, it appears that more research is necessary to examine this issue.

According to the results, the organic and functional groups had the lowest scores of conscientiousness. The scores of conscientiousness in neurogenic and vocally healthy groups were relatively close together. The depression scores were high in organic and functional groups. The highest scores of anxiety belonged to neurogenic and functional groups. As it was expected, functional dysphonic patients had to be more anxious than vocally organic dysphonic patients (Roy et al., 2000b). This result implied that the dysphonia type does have a relationship with personality traits and psychological distress, which is in line with the previous studies (Aldridge‐Waddon et al., 2023). Nevertheless, it may raise valid concerns regarding the significance of categorizing voice disorders in such a manner. The continuous debate revolves around the challenge of defining symptoms when there is an overlap between functional and organic manifestations of dysphonia, alterations in vocal or laryngeal perception, and/or voice problems in relation to upper airway complications like persistent cough (Aldridge‐Waddon et al., 2023).

Regarding the relationship between personality traits and psychological distress, it was found that participants with higher levels of extraversion, agreeableness, and conscientiousness experienced lower levels of anxiety, stress, and depression, while participants with higher levels of neuroticism had higher levels of depression, anxiety, and stress. Interestingly, only openness did not show any relationship with personality traits in the current study. One plausible reason for this result could be that the personality characteristic of openness might not have a direct impact on or correlation with the occurrence of depression, anxiety, or stress within this specific group. The results highlight the complex relationships between personality traits and psychological distress in individuals with normal voice and voice disorders, which are compatible well with other research done (Sancak et al., 2023).

Given the relatively high prevalence rate of voice disorders (16%–20%) and the significant economic burden they pose for governments, insurance, and individuals (annual costs ranging from $178,524,552 to $294,827,671) (Cohen et al., 2012), it appears effective to implement screening measures within society in terms of personality traits and psychological distress, and individuals identified as being at risk based on their conscientiousness scores should receive appropriate preventative training to address any incorrect vocal habits, under the guidance of SLPs. This approach is likely to lead to a reduction in the overall incidence of voice disorders within the population. Based on the results of this study, it seems a smart clinical choice to incorporate personality traits and psychological distress into voice therapy to enhance the care provided to dysphonic patients, including assessment and treatment. According to the findings of a systematic review, the inclusion of psychotherapeutic approaches such as CBT alongside traditional voice therapy may lead to improved treatment outcomes (Gray et al., 2021).

Despite personality traits being rooted in biology as dispositional or fundamental tendencies, it is important to acknowledge that culture can still have an impact on them. It is crucial to understand that, first, the universality of the five factors does not automatically imply the absence of personality traits unique to particular cultures, and, second, these five factors may not hold the same level of significance across all cultures (Keith, 2019). For instance, the results of our study highlighted the difference in conscientiousness between vocally healthy and dysphonic groups in the context of Persian culture, while in the research done in Hebrew culture, openness was found a significant personality trait being different between these two groups (Amir et al., 2023). Hence, it is recommended that subsequent studies investigate the reliability of these findings with other standardized psychological tests in different cultures and languages.

Limitations of this study should be regarded. First, the cross‐sectional design of the study hinders the investigation of causal interpretations. Second, it is notable that all the data discerning the personality traits and psychological distress were established by self‐report questionnaires; therefore, they are susceptible to probable self‐report bias. It is recommended that future studies will be designed as longitudinal and using clinical interviews, as well.

## 5 CONCLUSIONS

In conclusion, the findings showed that there is a potential relationship between personality and dysphonia by their effects on self‐control and self‐regulation processes. This may suggest the next research investigating the role of mediating variables of this relationship between personality traits with self‐control and self‐regulation processes. Dysphonic patients were different from vocally healthy people in conscientiousness as one of the main personality traits, and also in depression, and anxiety. Consequently, personality traits and psychological distress should be deemed in the prevention, evaluation, and treatment processes of voice disorders by voice care teams. The importance of access to and familiarity with short questionnaires measuring general personality and psychological characteristics, in voice clinics, cannot be overstated for preliminary screening. Patients’ referral to relevant mental health professionals is necessary if there were warning signs. Hence, it is recommended that in consultation with mental health professionals, such tools will be made.

## AUTHOR CONTRIBUTIONS

**Mahshid Aghajanzadeh**: Conceptualization; writing—review and editing; funding acquisition; supervision; project administration. **Payman Dabirmoghaddam**: Conceptualization; writing—review and editing; methodology; resources. **Mehdi Soleimani**: Writing—review and editing; methodology; formal analysis. **Saeed Saeedi**: Writing—review and editing; writing—original draft; investigation.

## FUNDING STATEMENT

This research received no specific grant from any funding agency in the public, commercial, or not‐for‐profit sectors.

## CONFLICT OF INTEREST STATEMENT

The authors declare no conflicts of interest.

### PEER REVIEW

The peer review history for this article is available at https://publons.com/publon/10.1002/brb3.3641.

## Data Availability

The corresponding author, Saeed Saeedi, can provide the data supporting the findings of this study upon request. The data are not accessible to the public as they contain sensitive information that could potentially jeopardize the privacy of the research participants and raise ethical concerns.
